# Clinical, Lab, and Radiological Evolution of an Adult Patient With Unilateral Cortical Lesion in Anti-Myelin Oligodendrocyte Glycoprotein (MOG)-Associated Encephalitis With Seizures and Anti-Glial Fibrillary Acidic Protein (GFAP) Positive Antibodies

**DOI:** 10.7759/cureus.70546

**Published:** 2024-09-30

**Authors:** Michail Papantoniou, Georgia Panagou, Konstantinos Kanavouras

**Affiliations:** 1 Neurology, General Hospital of Athens G. Gennimatas, Athens, GRC

**Keywords:** autoimmune encephalitis, case report, flames, gfap, mog

## Abstract

Myelin oligodendrocyte glycoprotein (MOG) antibody-associated disease refers to a clinical and radiological spectrum of demyelinating disorders of the Central Nervous System. We report the case of a female adult patient presenting to our department with an episode of seizures and cognitive dysfunction, compatible with Gerstmann syndrome. Brain MRI revealed a high T2 and DWI signal unilateral cortical lesion at the inferior left parietal lobe and leptomeningeal contrast enhancement. Lumbar puncture showed pleocytosis of the lymphocytic type and elevated protein. Upon suspicion of autoimmune encephalitis, extensive laboratory testing was performed, and the patient’s serum and cerebrospinal fluid (CSF) tested positive for anti-MOG, while anti-glial fibrillary acidic protein (GFAP) antibodies were detected in her serum. A diagnosis of FLAIR-hyperintense lesions in anti-MOG-associated encephalitis with seizures (FLAMES) was made, and the patient was treated with intravenous methylprednisolone for five days, leading to clinical remission within days. However, serum anti-MOG IgG titers were found to be higher on follow-up, and the patient experienced a relapse, thus treatment with azathioprine was initiated. We suggest that upon suspicion of autoimmune encephalitis, all patients should be tested for serum and CSF anti-MOG IgG antibodies. Furthermore, we consider that anti-MOG antibody titers and GFAP concentration could be used as possible biomarkers for the disease course and treatment strategy options.

## Introduction

Anti-myelin oligodendrocyte glycoprotein (MOG) disease is an autoimmune disorder in which the immune system mistakenly attacks MOG, a protein located on the surface of myelin, an insulating layer that surrounds nerve cell axons and enhances signal conduction between them [1]. The spectrum of demyelinating disorders with IgG antibodies to MOG, known as MOG antibody-associated disease (MOGAD), includes optic neuritis, encephalitis, myelitis, acute disseminated encephalomyelitis (ADEM), and other variants [2]. Over the last decade, progress in research and increasing information about the disorder have led to the hypothesis that MOGAD refers to a new entity, distinct from other demyelinating diseases of the central nervous system, such as multiple sclerosis (MS) and neuromyelitis optica (NMO) spectrum disorders. New insights into the disorder’s features and variants indicate a broader spectrum of the disease [1,2].

The aim of our paper is to report a rare case of the novel clinic-radiological sub-entity of MOGAD, FLAIR-hyperintense lesions in anti-MOG-associated encephalitis with seizures (FLAMES), and to provide our experience from this case to enrich the existing literature on the clinical, laboratory, and radiological aspects of MOGAD’s spectrum.

## Case presentation

**Figure 1:**
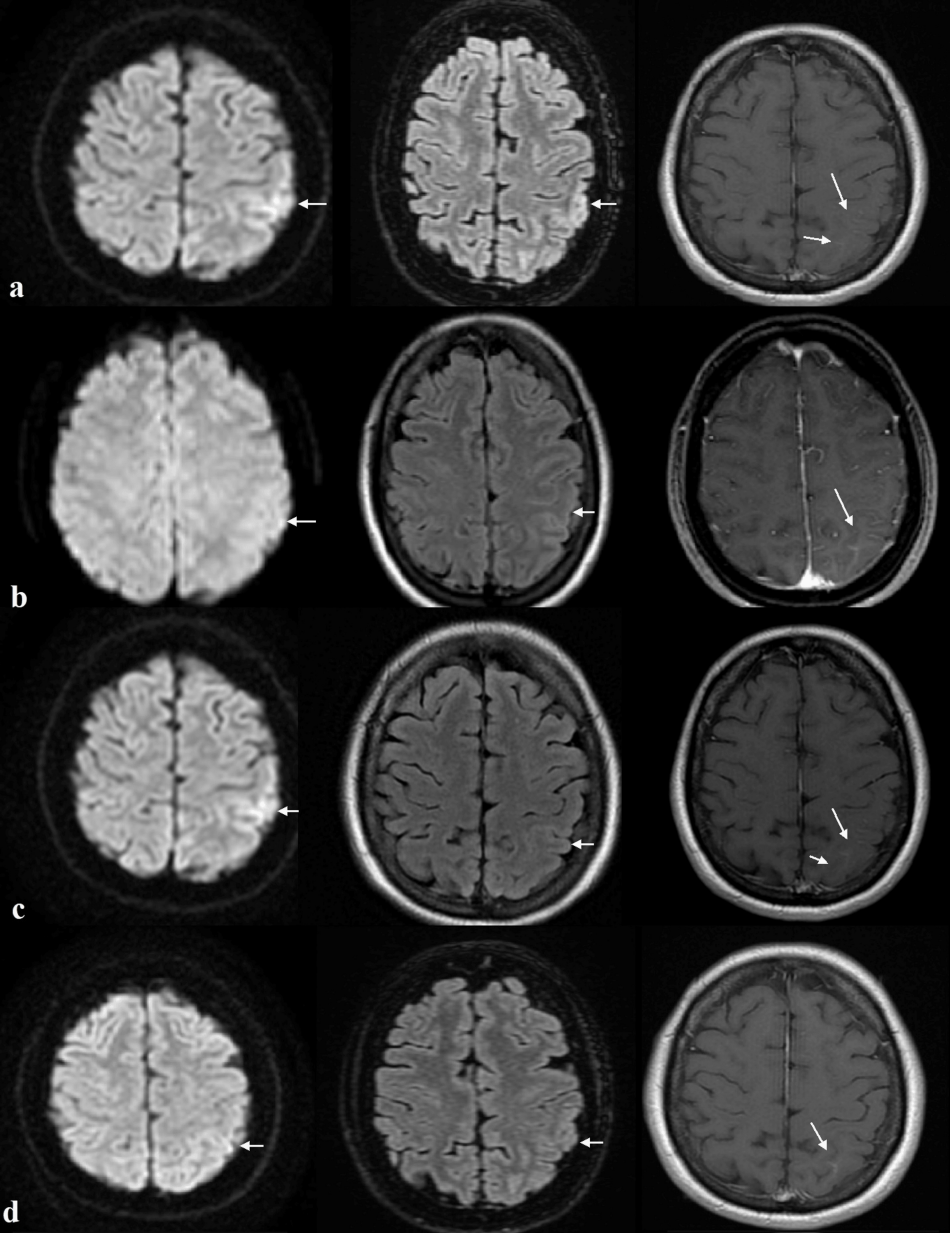
Axial MRI of the brain showing a high DWI and T2 signal unilateral cortical lesion at the left parietal lobe and leptomeningeal contrast enhancement (arrows). a. Performed nine days before her admission to our hospital; b. Two days after her admission to our hospital; c. During a relapsing episode, two months after the six-month follow-up; d. At the 12-month follow-up. Sequences from left to right: Diffusion-weighted imaging; Fluid-attenuated inversion recovery; T1+ Gadolinium.

A 48-year-old female patient was hospitalized in the Department of Neurology for two days, due to an episode of generalized tonic-clonic seizures lasting for two minutes. Prior to that, the patient had no history of epilepsy, head trauma, or any other neurologic or medical condition. The patient’s accompanying relatives and the patient herself reported no signs or symptoms of infection for the past nine months. The neurological examination was reported as unremarkable. A CT of the brain, an electroencephalogram (EEG), and a lumbar puncture (one cell per cubic millimeter, protein 36 mg/dL, and normal glucose value) had been performed, without abnormal findings. The patient was discharged without any medication but with a recommendation for a repeated EEG, brain MRI, and neurological reevaluation. A brain MRI had been performed three days after her discharge (Figure 1a) and had been reported as lacking abnormal findings; the patient only complained of a left parieto-temporal mild, pulsating headache since the day of her discharge.

Nine days later (14 days after her first seizure), the patient presented to our ED approximately 25 minutes after a similar episode of generalized seizures lasting for three minutes. The patient was afebrile, with normal vital signs, in a postictal state, disoriented in place and time, without any other noticeable neurological signs at that time. No structural abnormalities were reported on brain CT. A new lumbar puncture was performed, this time revealing mild pleocytosis (50 cells per cubic millimeter), mildly elevated protein (56 mg/dL), and normal glucose value. Polymerase chain reaction (PCR) testing of CSF for viral, bacterial, fungal, and parasitic infections was negative. Nevertheless, antiviral treatment with IV acyclovir as well as anticonvulsant treatment with levetiracetam 1000 mg twice daily was initiated.

**Figure 2:**
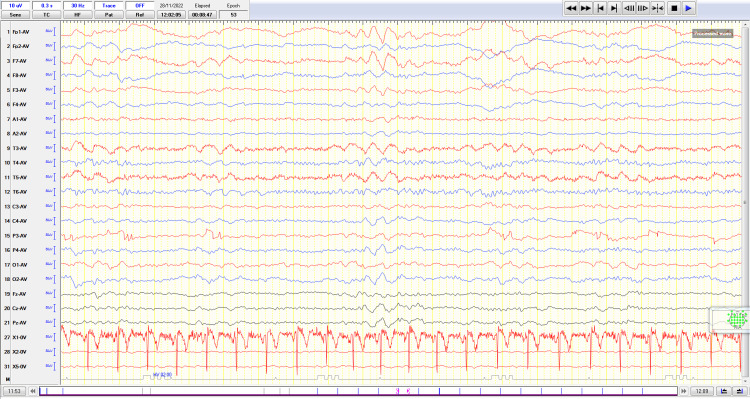
Electroencephalogram showing focal slow-wave activity (theta) in the left parietal-temporal region.

Eleven hours after her admission, the patient had fully recovered from her postictal confusion. Detailed examination of cognitive function, however, revealed dysgraphia, finger agnosia, acalculia, and left-right disorientation, compatible with Gerstmann syndrome [3], without any other neurological deficits. Brain MRI revealed a high T2 and diffusion-weighted imaging (DWI) signal unilateral cortical lesion at the left parietal lobe and leptomeningeal contrast enhancement (Figure 1b). Retrospectively, this unreported lesion was already visible in the patient’s first brain MRI. Consistent with the MRI, a new EEG also revealed focal slow-wave activity (theta) in the left parietal-temporal region (Figure 2).

Lumbar puncture was repeated five days later, showing further CSF pleocytosis (301 cells per cubic millimeter) of lymphocytic type, elevated protein (106 mg/dL), elevated IgG value (58.2 mg/L), and normal glucose value. PCR testing for viral, bacterial, fungal, and parasitic infections was again negative, as well as serum and CSF IgM and IgG antibodies against herpes simplex virus 1 & 2, varicella zoster virus, cytomegalovirus, West Nile virus, Epstein-Barr virus, enterovirus, adenovirus, influenza, echovirus, mumps, measles, rubella, Chlamydia pneumoniae, Mycoplasma pneumoniae, and Borrelia burgdorferi. Oligoclonal bands were absent in serum or CSF, and IgG Index was 0.55. Acyclovir was discontinued after 14 days of treatment, without any signs of clinical improvement up to that date.

Suspecting immune-mediated encephalitis, we expanded our investigation with serum and CSF testing for antibodies associated with autoimmune, demyelinating, and paraneoplastic encephalitides (voltage-gated potassium channel, contactin-associated protein-2 and leucine-rich glioma-inactivated 1, N-methyl-D-aspartate receptor, alpha-amino-3-hydroxy-5-methyl-4-isoxazolepropionate receptor1-2, glutamic acid decarboxylase, gamma-aminobutyric acid B receptor, MOG, aquaporin-4, anti-Hu, anti-Yo, anti-Ri, anti-CV2, anti-Ma2, anti-Tr, Zic4, and SOX1). The patient’s serum and CSF were found positive only for anti-MOG antibodies (cell-based assay - titers 1:40 and 1:16, respectively; abnormal values: ≥ 1:20 and 1:16, respectively). Subsequently, the patient’s samples were tested for anti-glial fibrillary acidic protein (GFAP) antibodies; her serum was positive (titer 1:32), her CSF negative. Spinal MRI and optical coherence tomography (OCT) were performed, without abnormal findings. Based on the clinical, radiological, and laboratory findings, a diagnosis of FLAMES was made. The patient was treated with high-dose IV methylprednisolone (1000 mg per day) for five days, followed by a calculated oral prednisone dosage (1 mg/kg per day), tapered slowly over 12 weeks, which led to complete remission of Gerstmann syndrome within a few days. Upon request, a CSF sample was provided to us, kept in a -80° C freezer from the patient’s previous hospitalization (no serum sample had been stored), and was tested for anti-MOG and anti-GFAP antibodies, but was found negative.

At six-month follow-up, her neurological examination was normal, and the patient reported no relapses of neurological symptoms or seizures. A repeated lumbar puncture was normal (zero cells per cubic millimeter, protein 25 mg/dL, and normal glucose value). Serum and CSF anti-MOG and anti-GFAP antibodies were also tested, and only the patient’s serum was found positive for antibodies against MOG (titer 1:80). No further immunosuppressive treatment was administered to the patient.

Two months later, the patient presented to us with a relapsing episode of aphasia. Repeated brain MRI showed no significant changes, except a higher extent and intensity of the abnormal DWI signal lesion (Figure 1c). CSF tests were normal, but serum anti-MOG antibodies were found again positive with an increased titer (1:160); thus, the patient was treated with high-dose IV methylprednisolone for three days, after which complete clinical remission was noted. Subsequently, immunosuppressive treatment with azathioprine, gradually titrated up to 100 mg daily, was initiated. Serum GFAP concentration was measured at that time (according to our patient’s age and BMI, using the Simoa detection technique) and was found to be 119.0 pg/mL (healthy control reference <60.1 pg/mL).

Three months later, the patient was reevaluated, and no relapses were reported. Repeated brain MRI showed reduced extent and intensity of the abnormal T2 and DWI signal lesions and minimal contrast enhancement (Figure 1d). Serum anti-MOG antibodies remained positive, but at a lower titer (1:40), and immunosuppressive treatment was continued.

## Discussion

Herein, we present a 12-month history of an adult patient presenting with seizures, a unilateral cortical MRI lesion at the left parietal lobe, CSF pleocytosis and elevated protein, anti-MOG and anti-GFAP IgG antibodies, as well as a focal neurological deficit (Gerstmann syndrome) attributable to the cortical lesion, indicating the diagnosis of autoimmune encephalitis. Our patient met the newly proposed diagnostic criteria for MOGAD [4] and specifically the FLAMES variant [5], while other possible causes were excluded, as demonstrated below.

CSF pleocytosis with lymphocytic predominance (range 3-306) and elevated protein is common in MOGAD, whereas oligoclonal bands are typically absent in CSF, and IgG Index values are found to be < 0.6, due to possible peripheral synthesis of anti-MOG IgG, as seen in our case [6-8]. Moreover, the patient reported no symptoms or clinical signs of any kind of infection for at least nine months prior to admission to our hospital. Brain MRI showed unilateral cortical lesions, in contrast to bilateral lesions with ill-defined borders, which typically appear in ADEM-MOG encephalomyelitis [9]. Infectious disease was excluded through the clinical course and negative CSF cultures and PCR, while MS and neuromyelitis optica spectrum disorder (NMOSD) were ruled out by means of laboratory and radiological findings: CSF oligoclonal bands and anti-aquaporin-4 IgG antibodies were negative, OCT was normal, and MRI of the brain and the spinal cord revealed neither white matter lesions suggestive of MS, nor lesions of the optic nerves, the area postrema, the medulla, or the spinal cord [9].

The coexistence of MOG and GFAP IgG antibodies has been described before in three cases, each regarded as a possible overlapping syndrome with distinct pathogenesis [10-12]. We consider that the finding of concurrent anti-MOG and GFAP antibodies in our patient does not represent a different pathogenesis, but more likely a secondary cross-reactive immune response upon antigen presentation, due to astrocytic damage. Furthermore, anti-GFAP antibodies showed a remarkable response to steroid treatment and were not detected later in the disease’s course, consistent with the data we found in the literature [13]. In contrast, serum GFAP concentration can remain elevated even after steroid treatment and may be used as a biomarker for the disease course, as seen in our patient [13,14].

FLAMES refers to a rare, novel clinic-radiological entity of MOGAD, characterized by unilateral cortical lesions, MOG-IgG antibodies, seizures, and other neurological deficits. CSF samples usually show pleocytosis and elevated proteins, while MRI of the brain reveals high T2 signal cortical lesions, usually with high signal on DWI and leptomeningeal enhancement. The management and prognosis of FLAMES do not differ from other MOGAD phenotypes. Treatment includes IV methylprednisolone, plasma exchange, IV immunoglobulin, or cyclophosphamide, and fewer or no relapses are reported, compared with MS and NMOSD [5,15,16]. In our patient, IV methylprednisolone led to the complete remission of her deficits until after her 6-month follow-up. Nonetheless, a clinical relapse occurred eight months after the first episode, and in addition, persistent, rising titers of serum anti-MOG IgG antibodies were detected. Thus, the initiation of immunosuppressive treatment with azathioprine was decided upon, considering that our patient now had a high risk of relapse [17,18].

## Conclusions

In this case report, we present the 12-month clinical, radiological, and laboratory evolution of a patient with a rare case of FLAMES, positive anti-GFAP antibodies, and Gerstmann syndrome. Our case indicates that, upon suspicion of autoimmune encephalitis with clinical or neuroradiological findings indicating cortical involvement, testing for anti-MOG IgG antibodies in serum and CSF is warranted. Coexisting positive testing for anti-GFAP antibodies should not alter the diagnosis, likely representing astrocytic damage. Furthermore, our case emphasizes the need for long-term clinical and radiological follow-up in FLAMES, comparable to other, more common MOGAD phenotypes. Lastly, we suggest that anti-MOG antibody titers and GFAP concentration could be used as possible biomarkers for the disease course and treatment strategy options.

## References

[1] Reindl M, Rostasy K (2015). MOG antibody-associated diseases. Neurol Neuroimmunol Neuroinflamm.

[2] Dos Passos GR, Oliveira LM, da Costa BK, Apostolos-Pereira SL, Callegaro D, Fujihara K, Sato DK (2018). MOG-IgG-associated optic neuritis, encephalitis, and myelitis: lessons learned from neuromyelitis optica spectrum disorder. Front Neurol.

[3] Ardila A (2020). Gerstmann syndrome. Curr Neurol Neurosci Rep.

[4] Kim KH, Kim SH, Park NY, Hyun JW, Kim HJ (2023). Validation of the International MOGAD Panel proposed criteria. Mult Scler.

[5] Jain K, Cherian A, Divya KP, Rajalakshmi P, Thomas B, Nandana J (2021). FLAMES: a novel burning entity in MOG IgG associated disease. Mult Scler Relat Disord.

[6] Jarius S, Ruprecht K, Kleiter I (2016). MOG-IgG in NMO and related disorders: a multicenter study of 50 patients. Part 1: frequency, syndrome specificity, influence of disease activity, long-term course, association with AQP4-IgG, and origin. J Neuroinflammation.

[7] Jarius S, Ruprecht K, Kleiter I (2016). MOG-IgG in NMO and related disorders: a multicenter study of 50 patients. Part 2: Epidemiology, clinical presentation, radiological and laboratory features, treatment responses, and long-term outcome. J Neuroinflammation.

[8] Kitley J, Waters P, Woodhall M (2014). Neuromyelitis optica spectrum disorders with aquaporin-4 and myelin-oligodendrocyte glycoprotein antibodies: a comparative study. JAMA Neurol.

[9] Shahriari M, Sotirchos ES, Newsome SD, Yousem DM (2021). MOGAD: How it differs from and resembles other neuroinflammatory disorders. AJR Am J Roentgenol.

[10] Ding J, Ren K, Wu J, Li H, Sun T, Yan Y, Guo J (2020). Overlapping syndrome of MOG-IgG-associated disease and autoimmune GFAP astrocytopathy. J Neurol.

[11] Ji S, Liu C, Bi Z, Gao H, Sun J, Bu B (2021). Overlapping syndrome mimicking infectious meningoencephalitis in a patient with MOG and GFAP IgG. BMC Neurol.

[12] Martin AJ, Strathdee J, Wolfe N (2022). Coexistent anti-GFAP and anti-MOG antibodies presenting with isolated meningitis and papillitis: more support for overlapping pathophysiology. BMJ Neurol Open.

[13] Yang X, Huang Q, Yang H (2019). Astrocytic damage in glial fibrillary acidic protein astrocytopathy during initial attack. Mult Scler Relat Disord.

[14] Mader S, Kümpfel T, Meinl E (2022). Pathomechanisms in demyelination and astrocytopathy: autoantibodies to AQP4, MOG, GFAP, GRP78 and beyond. Curr Opin Neurol.

[15] Budhram A, Kunchok A, Flanagan E (2020). Adding FUEL to the FLAMES: FLAIR-variable unilateral enhancement of the leptomeninges (FUEL) in MOG-IgG-associated disease. Neurology.

[16] Parrotta E, Kister I (2020). The expanding clinical spectrum of myelin oligodendrocyte glycoprotein (MOG) antibody associated disease in children and adults. Front Neurol.

[17] López-Chiriboga AS, Majed M, Fryer J (2018). Association of MOG-IgG serostatus with relapse after acute disseminated encephalomyelitis and proposed diagnostic criteria for MOG-IgG-associated disorders. JAMA Neurol.

[18] Hennes EM, Baumann M, Lechner C, Rostásy K (2018). MOG spectrum disorders and role of MOG-antibodies in clinical practice. Neuropediatrics.

